# Improving “reasonable adjustments” for people with autism in the York Early Intervention in Psychosis Service

**DOI:** 10.1192/bjo.2021.941

**Published:** 2021-06-18

**Authors:** Daniel Whitney, Emma Faravelli, Stephen Wright

**Affiliations:** Tees Esk and Wear Valleys NHS Foundation Trust

## Abstract

**Aims:**

Studies show the prevalence of Autism Spectrum Conditions in EIP populations is 3.6-3.7% compared to approximately 1-1.5% in the general population. The Equality Act 2010 and the Autism Act 2009 make it a requirement for services to make ‘Reasonable Adjustments’ for people with Autism. The aim of this study was to improve how our service makes Reasonable Adjustments for people with autism.

**Method:**

There were 15 patients in our service with a confirmed diagnosis of Autism. Pre and Post a discussion about reasonable adjustments, we invited them to rate, on a 5 point Likert scale, how well they felt the service was making Reasonable Adjustments for their Autism and whether discussing it had been helpful. We offered face to face or telephone discussions with someone with autism expertise to discuss reasonable adjustments. We allowed at least a month after the discussion before repeating the Likert scale.

**Result:**

The pre-discussion rating, of whether the team was making reasonable adjustments for Autism, showed agreement (mean 4.2/5). This improved to 4.6/5 after a month post discussion about reasonable adjustments. Patients agreed to strongly agreed (4.6/5) that the discussion had been helpful. Reasonable adjustments identified were quite individual but responses followed the following main themes; (1) No adjustments were needed or wanted as some patients saw special arrangements for them as stigmatising and wanted to be treated like everyone else; (2) Adjustments around personal space in appointments eg sitting face to face, not sitting too close, explaining reason before moving closer; (3) Simplification/clarification of written information – eg some identified simpler language use and use of pictures; (4) Environment e.g. quieter, dimmed lights, clarity of signage in reception.

**Conclusion:**

Autistic patients in our service already rated the team highly at making reasonable adjustments pre and post intervention and found it helpful to have a specific discussion. Reasonable adjustments were highly individualised but some themes emerged around personal space, written communication and clinic environment which staff could consider exploring routinely. Some patients did not want reasonable adjustments as they felt it could be stigmatising. Discussing reasonable adjustments is likely to benefit all patients, not just those with confirmed autism, we would suggest this should be built into routine practice.

